# *In vitro* stimulation of calcium overload and apoptosis by sonodynamic therapy combined with hematoporphyrin monomethyl ether in C6 glioma cells

**DOI:** 10.3892/ol.2014.2419

**Published:** 2014-08-05

**Authors:** SHAOCHUN DAI, CHANGQING XU, YE TIAN, WEN CHENG, BO LI

**Affiliations:** 1 Department of Ultrasound, The Third Affiliated Hospital, Harbin Medical University, Harbin, Heilongjiang 150081, P.R. China; 2 Department of Pathophysiology, Harbin Medical University, Harbin, Heilongjiang 150081, P.R. China

**Keywords:** C6 glioma cells, sonodynamic therapy, apoptosis, calcium overload, reactive oxygen species

## Abstract

The present study investigated enhancement of apoptosis induction and the mechanisms underlying calcium overload on C6 glioma cells *in vitro,* stimulated by low-level ultrasound in combination with hematoporphyrin monomethyl ether (HMME). The optimum frequency of ultrasound was determined by 3-(4,5-dimethythiazol-2-yl)-2,5-diphenyltetrazolium bromide assay. The apoptotic rate, reactive oxygen species concentration and decreased mitochondrial membrane potential (MMP) were analyzed by flow cytometry. Morphological changes were detected by a transmission electron microscope, and the concentration of intracellular Ca^2+^, [Ca^2+^]_i_, was detected by a confocal laser scanning microscope. In addition, the release of cytochrome *c* (cyt-*c*) was measured by western blot analysis. The results revealed that an increased apoptotic effect was induced by sonodynamic therapy (SDT), and this was found to correlate with the overloaded [Ca^2+^]_i_, derived from the intra- and extracellular sources in the early apoptotic process. The results also revealed an increased level of ROS production, a decreased MMP and an increased release of cyt-*c*. The present study indicated that low-level ultrasound in combination with HMME improved the apoptotic effect in C6 glioma cells. The overloaded [Ca^2+^]_i_ was involved in the mechanism by which apoptosis was stimulated and enhanced by SDT.

## Introduction

Due to the severely infiltrative nature of glioma and the resulting difficulty in performing complete surgical resection, there is often a high rate of recurrence and metastasis in glioma patients undergoing surgical treatment alone. Radiotherapy, chemotherapy and photodynamic therapy exhibit certain auxiliary functions for the gliomas, however, they are insufficient according to the published median survival analysis (1). Sonodynamic therapy (SDT) is a novel therapy for tumor treatment, which focuses the ultrasound energy on malignant sites located deeper in the tissues, which cooperate with sonosensitizers, and thus exert significant antitumor effects *in vitro* and *in vivo*. Previous studies have indicated that ultrasound in combination with porphyrins may induce apoptosis in glioma cells (2–6). However, a relatively low apoptotic rate has been identified following SDT (3–6). Previous studies have indicated that lower frequency and power may lead to a higher cavitation effect and concomitant biological effect (7–9). For satisfactory preferential retention and a long term storage of hematoporphyrin monomethyl ether (HMME) in glioma tissue, in this study, the C6 glioma cells were treated with HMME and lower level ultrasound using optimal parameters to enhance the apoptotic effect *in vitro*.

Apoptosis is regulated by numerous factors, including reactive oxygen species (ROS), calcium overload and mitochondrial damage (10–12). The calcium ion has been proposed to be an important regulator in apoptosis. A rapid increase in intracellular Ca^2+^, [Ca^2+^]_i_, has been identified in various conditions, including ischemia-reperfusion injury, receptor over-stimulation and oxidative stress, among others (13–16). Calcium overload may damage mitochondria, causing the release of apoptotic promoters and activation of the caspase cascade (17,18). Honda *et al* (17) found a transient increase in [Ca^2+^]_i_ from outside the cells in human myelomonocytic lymphoma U937 cells following ultrasound alone, and Li *et al* (18) showed an increased [Ca^2+^]_i_, released from internal stores at an ultrasound frequency of 1 MHz in combination with HMME in C6 glioma cells (18). This may be attributed to the ultrasonic cavitation effect, which changes the permeability of the cytomembrane (19,20). Therefore, overloaded [Ca^2+^]_i_ may be obtained from internal and external sources during the apoptotic process in C6 glioma cells, as a result of low-level ultrasound and HMME. In the present study, the source of overloaded [Ca^2+^]_i_. was detected from intracellular and extracellular environments following SDT.

Previous studies have demonstrated the SDT may induce apoptosis in C6 glioma cells via the excessive production of ROS, which was due to the interaction of the ultrasonic cavitation and sensitizers (7–9,19,20). The oxidizing effect may damage mitochondria and lead to apoptosis via the mitochondrial signaling pathway (10–16). In addition, ROS increases cytosolic calcium in the absence of extracellular calcium, leading cells into an apoptotic state (17). Cavitations including inertial and stable cavitation, are associated with a number of biological process, including the production of free radicals, changes in membrane permeability and sonoluminescence, among others (14–17). Although the mechanism of ROS production is not clear, the cavitation effect must be involved in the apoptotic process in SDT and may be relevant to the overloaded Ca^2+^ and mitochondrial damage.

Accordingly, in this study we hypothesized that low-level ultrasound in combination with HMME may increase the apoptotic rate and the concentration of [Ca^2+^]_i_ in C6 glioma cells following SDT-HMME treatment, which is associated with ROS production, decreased mitochondrial membrane potential (MMP) and the release of cytochrome *c* (cyt-*c*). The apoptotic rate and concentration of [Ca^2+^]_i_ was determined following SDT-HMME treatment. The L-type Ca^2+^ channel antagonist nimodipine was added to cells prior to SDT to detect the source of overloaded [Ca^2+^]_i_.

## Materials and methods

### Reagents

The C6 glioma cell line was purchased from the Beijing Institute of Biology, Chinese Academy of Sciences (Beijing, China) and 2,7-dichlorodihydrofluorescein diacetate (DCFH-DA), Rhodamine 123 and fluo-3/acetoxymethylester were purchased from Sigma-Aldrich (St. Louis, MO, USA). Fluorescein isothiocyanate (FITC)-Annexin-V/propidium iodide (PI) and Hank’s balanced salt solution (HBSS) were purchased from Beyotime Institute of Biotechnology (Jiangsu, China) and 3-(4,5-dimethythiazol-2-yl)-2,5-diphenyltetrazolium bromide (MTT) was purchased from Sigma-Aldrich. Nimodipine was purchased from Bayer (Leverkusen, Germany). HMME was purchased from Changzhou Kangmei Chemical Co., Ltd. (Jiangsu, China) and rabbit monoclonal anti-rat cyt-*c* antibodies were purchased from Santa Cruz Biotechnology, Inc. (Santa Cruz, CA, USA).

### Cell culture

The C6 glioma cells were cultured in RPMI-1640 medium (Hyclone Laboratories, Inc., Logan, UT, USA) containing 10% fetal bovine serum (Hyclone Laboratroies, Inc.). The cells were maintained at 37°C in a humidified atmosphere containing 5% CO_2_. One day prior to treatment, the cells were trypsinized, counted and seeded in six-well plates at a density of 1×10^6^/ml cells per well. Cells were cultured to 70–80% confluence prior to further experiments.

### Ultrasound frequency optimization

To optimize the ultrasound frequency, the cell viability was investigated by MTT assay as described previously (3,17). Cells were cultured at 37°C in six-well plates at a density of 1×10^6^/ml cells per well. The ultrasound irradiation was carried out at room temperature in a sponge water bath (depth, 10 mm) using a multi-function ultrasound device (ultrasound transducer diameter, 20 mm; depth of penetration, 50 mm; MB-200F, Saifuruide (Beijing) Technology Co., Ltd., Beijing, China), the device was customised by the College of Underwater Acoustic Engineering, Harbin Engineering University (Harbin, China), the frequency of the device was enabled to alter between 0.3 and 1.0MHz and the power could be adjusted from 0 to 1.0W. The sponge was placed under the wells, and the probe was placed under the sponge. The sponge water bath aided the minimization of acoustic reflections and subsequent standing wave formations. The pulsed-wave ultrasound parameters were set at 1 W/cm^2^ for intensity and 60 sec for duration time. The frequencies varied between 0 and 1.0 MHz. Cells were trypsinized and transferred to 96-well plates following irradiation. MTT was added to a final concentration of 0.5 mg/ml. Following 4 h of culture at 37°C, the supernatant was removed, and 200 μl dimethylsulfoxide (Sigma-Aldrich) was added. The absorbance was read at a wavelength of 490 nm using a universal microplate spectrophotometer (Model 550; Bio-Rad, Hercules, CA, USA). The cell viability without irradiation was considered as a control for 100% viability, and thus cell viability was expressed as a percentage of the control. Cell viability was statistically analyzed to select the appropriate frequency for further ultrasound experiments.

### SDT treatment

The ultrasound and SDT treatments for the C6 glioma cells were performed as previously described (3). Briefly, cells cultured at 37°C in six-well plates were randomly divided into control (untreated), HMME (HMME alone), ultrasound (ultrasound alone) and SDT (ultrasound + HMME) groups. Each group was placed in six wells. Cells in the four groups were pretreated with phosphate-buffered saline (PBS, Ca^2+^-free), HBSS (containing 1.3 mM Ca^2+^, pH 7.4), nimodipine (10 mg/ml in PBS) and HBSS-nimodipine for the follow-up experiments. HMME was added to the HMME and SDT group at a final concentration of 10 μg/ml for 2 h prior to insonation. Then cells in the four different groups were treated for an instant insonation. The ultrasound treatment was carried out under the same intensity and time, however, the frequency was determined by MTT assay. Following treatment, cells from the sixteen groups were maintained at 37°C in a humidified atmosphere containing 5% CO_2_ in the dark for subsequent experiments.

### Measurement of [Ca^2+^]_i_

The concentration of [Ca^2+^]_i_ was measured using a confocal laser scanning microscope (Leica TCS SP5; Leica, Mannheim, Germany) as described previously (18). Cultured C6 glioma cells in 96-well plates were loaded with 10 μM fluo-3/acetoxymethylester for 30 min at 37°C prior to SDT. Cells in the different groups were then washed three times with PBS to remove the extracellular fluo-3/acetoxymethylester for SDT. Excitation was set at a wavelength of 488 nm and emission was monitored at a wavelength of 530 nm. Fluorescence images indicating the [Ca^2+^]_i_ were captured using a confocal laser scanning microscope for 30 min (Leica).

### Detection of apoptosis

Cell apoptosis was measured by flow cytometry with double staining of FITC-Annexin-V and PI as previously described in the groups as determined by the previous confocal laser scanning assay (11). After 24 h, cells with and without treatment were harvested, washed twice with PBS and re-suspended with 0.5 ml PBS at a cell density of 1×10^6^ cells/ml. Next, a total of 10 μl Annexin-V and 5 μl PI were added to the wells in the dark. Following 30 min of incubation, the cells were analyzed by flow cytometry (Becton Dickinson, Franklin Lakes, NJ, USA) to determine the apoptotic rate.

### Transmission electron microscopy

After 24 h cells in the groups with or without treatment were harvested and fixed with 3.0% glutaraldehyde and 1.5% paraldehyde, washed in PBS, and fixed in osmium tetroxide. Then, cells were dehydrated in an ethanol series, embedded in epoxy resin and examined under a transmission electron microscope [JEM-1220EX; Jeol (GmbH), München, Germany].

### Production of intracellular ROS

The production of intracellular ROS was assayed by flow cytometry using DCFH-DA as previously described (11). After 2 h, cells in the groups with and without treatment were harvested, washed and re-suspended in 500 μl PBS containing DCFH-DA (final concentration, 10 mol/l), and then incubated at 37°C in the dark for 30 min. The cell analysis was performed by flow cytometry.

### MMP detection

The loss of MMP was detected by flow cytometry using Rhodamine 123 as previously described (11). After 2 h, Rhodamine 123 was added to the cells at a final concentration of 200 nmol/l in the dark and incubated for 30 min. The decrease in MMP was calculated using CellQuest software (BD Biosciences, Franklin Lakes, NJ, USA).

### Detection of cyt-c release from mitochondria

To quantify cyt-*c* release, western blot analysis of cyt-*c* in the cytosolic fraction was performed as previously described (11,21). After 24 h, cells were harvested, washed twice with ice-cold PBS, and incubated in an ice-cold Tris-sucrose buffer (0.35 M sucrose, 10 mM Tris-HCl, pH 7.5, 1 mM EDTA, 0.5 mM dithiothreitol and 0.1 mM phenylmethylsulfonyl fluoride). Following a 40-min incubation, cells were centrifuged at 1,000 × g for 5 min at 4°C, and the supernatant was further centrifuged at 40,000a × g for 30 min at 4°C. The supernatant was regarded as the cytosolic fraction, and resolved on SDS-PAGE gel and analyzed by western blot analysis using a primary rabbit monoclonal anti-rat antibody against cyt-*c* and a secondary goat polyclonal immunoglobulin G anti-rabbit IgG (Santa Cruz Biotechnology, Inc.). Actin expression was used as the loading control.

### Statistical analysis

Data are expressed as the mean ± standard error of the mean. Comparisons between the different groups were performed via factorial design analysis of variance using SPSS, version 11.0 (SPSS, Inc., Chicago, IL, USA). P<0.05 was considered to indicate a statistically significant difference.

## Results

### Optimal parameters for ultrasound application

**Figure 1:**
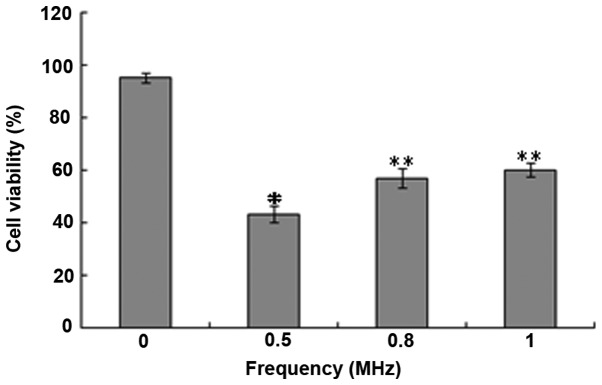
The viability rate of C6 glioma cells following ultrasound treatment at an intensity of 1.0 W/cm, for a duration of 60 sec, using various frequencies between 0 and 1.0 MHz. The viability rate decreased significantly at the ultrasound frequency of 0.5 MHz. ^*^P<0.05, vs. the other groups, and ^**^P<0.05, vs. the 0 and 0.5 MHz groups. Data are expressed as the mean ± SEM from six independent experiments.

Cells were exposed to pulsed-wave ultrasound with an irradiation time of 60 sec and an average intensity of 1.0 W/cm^2^. The optimum experimental frequency was determined by MTT assay according to the cell viability. As shown in Fig. 1, the survival rates were 95.4±1.8, 43.2± 3.2, 57.1± 3.7 and 60.2±2.6% at the frequencies of 0, 0.6, 0.8 and 1 MHz, respectively, following insonation. The survival rate was decreased significantly in the groups at the frequency of 0.5 MHz. In order to improve the apoptotic rate, 0.5 MHz was determined as the optimal frequency for the C6 glioma cells.

### Measurement of [Ca^2+^]_i_

**Figure 2:**
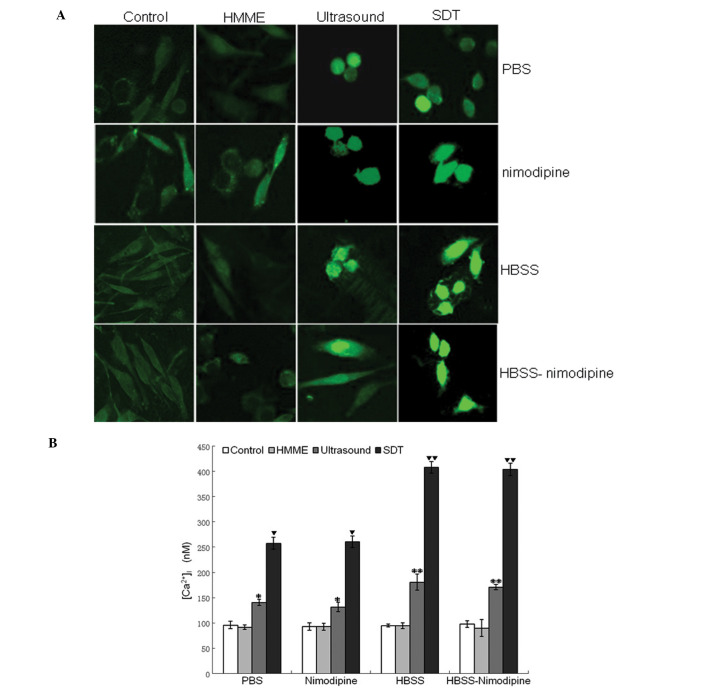
Changes in the concentration of the intracellular free calcium following SDT were investigated. (A) Fluorescent images of C6 glioma cells incubated in PBS, nimodipine, HBSS and HBSS-nimodipine solution were captured by a confocal laser scanning microscope in 1800s. (B) Changes in [Ca^2+^]_i_ concentration in the different groups were recorded in 1800s following SDT. The SDT-HBSS group showed the highest increase in [Ca^2+^]_i_ concentration. Mean values without the same symbol differ significantly (P<0.05). SDT, sonodynamic therapy; HBSS, Hank’s balanced salt solution; HMME, hematoporphyrin monomethyl ether; PBS, phosphate-buffered saline.

The concentration of intracellular free calcium was recorded by a confocal laser scanning microscopy for 30 min in a single cell with the fluorescent probe fluo-3/acetoxymethylester, a sensitive Ca^2+^ probe. The concentration of [Ca^2+^]_i_ was increased significantly in the ultrasound group with PBS, HBSS, nimodipine and HBSS-nimodipine solution, and further increased in the SDT groups with the same medium (P<0.05 versus the control-PBS group; 96±7.3 nM; Fig. 2A and B). This revealed that the overloaded [Ca^2+^]_i_ was involved in SDT treatment. Although SDT-PBS treatment significantly increased the concentration of [Ca^2+^]_i_ in 1,800s (258±11.8 nM; P<0.05 versus the control-PBS group), a higher elevation of [Ca^2+^]_i_ was observed in the SDT-HBSS group (408±11.6 nM; P<0.05 versus the other groups). In addition, no significant difference was identified in the elevated concentration of [Ca^2+^]_i_ between the SDT-HBSS-nimodipine (404±12.1 nM) and the SDT-HBSS group (408±11.6 nM) (P>0.05), which was the same as in the ultrasound-HBSS-nimodipine (181±16.2 nM) and the ultrasound-HBSS group (171±5.5 nM) (P>0.05).

### Apoptotic effect

**Figure 3:**
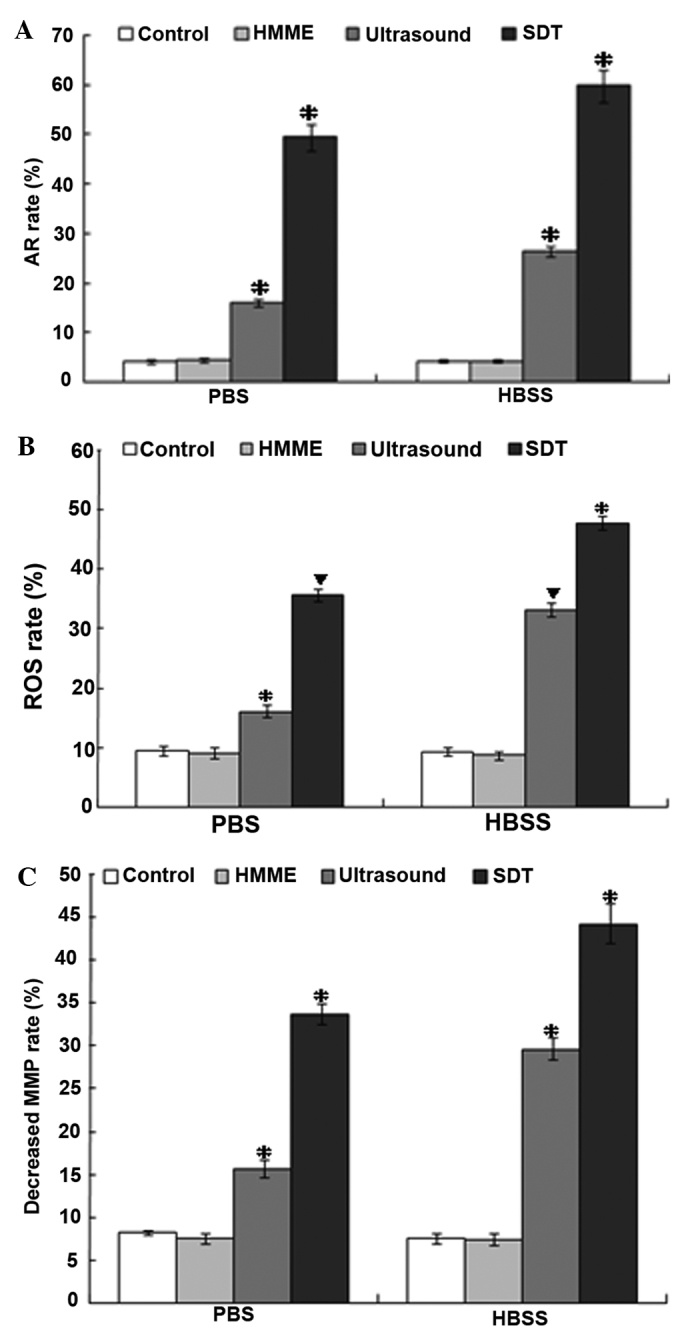
Apoptotic rate, ROS production and decreased MMP of C6 glioma cells from SDT, ultrasound, HMME and control groups in PBS and HBSS groups were analyzed by flow cytometry. (A) The apoptotic rate was increased the most in the SDT-HBSS group. (B) The production of ROS increased the most in the SDT-HBSS group. (C) The MMP decreased most in the SDT-HBSS group. ^*^P<0.05, vs. the other groups. ^▼^P<0.05, vs. the other groups except the group with the same symbol (^▼^). HMME, hematoporphyrin monomethyl ether; PBS, phosphate-buffered saline; HBSS, Hank’s balanced salt solution; MMP, mitochondrial membrane potential; ROS, reactive oxygen species.

To investigate the association between apoptosis and overloaded [Ca^2+^]_i_, apoptotic rates were determined in groups with overloaded [Ca^2+^]_i_ by flow cytometry. Cells were divided into control, HMME, ultrasound and SDT groups in PBS and HBSS solutions. The SDT and ultrasound groups in the two types of solution exhibited a significant increase in the apoptotic rate (P<0.05, versus the control-PBS group; 4.2±0.5%; Fig. 3A). Ultrasound-HBSS (26.5±1.1%) and ultrasound-PBS (16.0±0.8%) treatment exhibited a marginal apoptotic effect (P<0.05, versus the control-PBS group) (17). SDT-PBS treatment (49.4±2.6%) exhibited a significant apoptotic effect (P<0.05, versus the other groups) and SDT-HBSS treatment (59.9±3.2%) exhibited the highest rate (P<0.05, versus the other groups).

### Intracellular ROS

The production of ROS was determined by flow cytometry with DCFH-DA. Intracellular ROS significantly increased, with the exception of the control and HMME groups in PBS and HBSS (Fig. 3B). The production of increased ROS in the ultrasound-PBS, ultrasound-HBSS, SDT-PBS and SDT-HBSS groups were 16.1±1.0, 33.1±1.1, 35.6±1.0 and 47.7±1.2%, respectively. This increase was concurrent with the increase in apoptotic rate and overloaded [Ca^2+^]_i_ in the same group following SDT treatment (P<0.05, versus the control-PBS group; 9.4±0.9%; Fig. 3B). However, no significant difference in ROS production was identified between the ultrasound-HBSS and the SDT-PBS groups (P>0.05).

### Loss of MMP

Cells undergoing apoptosis usually lose their MMP and appear Rhodamine 123-dim. Thus, MMP was determined by Rhodamine 123 staining and detected by flow cytometry. The MMP decreased significantly in the ultrasound-PBS (15.7±1.0%) and ultrasound-HBSS (29.6±1.3%) groups (P<0.05, versus the control-PBS group; 8.2±0.3%); Fig. 3C). Furthermore, the MMP decreased more clearly in the SDT-PBS (33.7±1.2%) group and was the largest in the SDT-HBSS group (44.2±2.3%). The decrease in MMP in the groups was similar to that of the increased ROS and the overloaded [Ca^2+^]_i_ in the apoptotic process by SDT.

### Release of cyt-c

**Figure 4:**
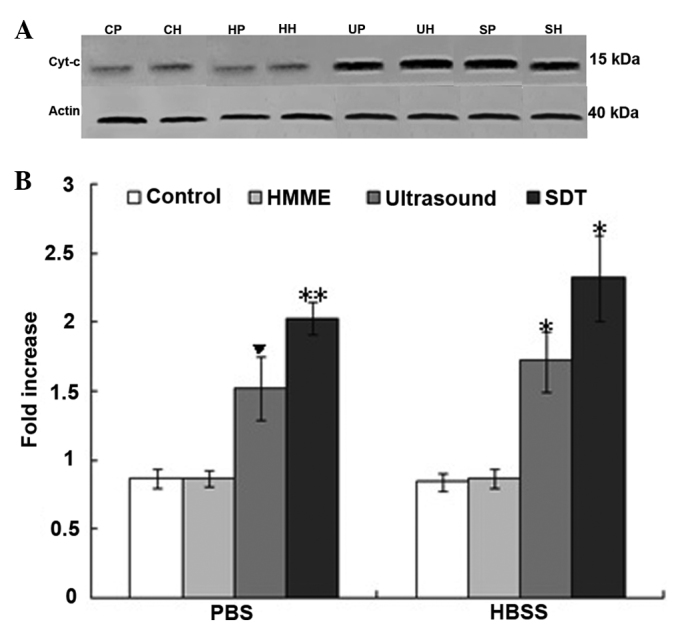
(A) The release of cyt-*c* from the control, HMME, ultrasound and SDT groups in PBS and HBSS in C6 glioma cells from the control-PBS (CP), HMME-PBS (HP), ultrasound-PBS (UP), SDT-PBS (SP), control-HBSS (CH), HMME-HBSS (HH), ultrasound-HBSS (UH) and the SDT-HBSS (SH) groups were analyzed using western blot analysis. (B) Upregulation of cyt-*c* release was exhibited in the UP, SP, UH and SH groups. ^▼^P<0.05 vs. the other groups; ^*^P<0.05, vs. the other groups, with the exception of the SP group; and ^**^P<0.05, vs. the other groups, with the exception of the UH and SH groups. Cyt-*c*, cytochrome *c*; HMME, hematoporphyrin monomethyl ether; PBS, phosphate-buffered saline; HBSS, Hank’s balanced salt solution.

Since the MMP was disrupted following SDT treatment, the modulation of the expression of key signaling molecules of the mitochondrial signaling pathway under low-level ultrasound and HMME was investigated. The release of cyt-*c* was measured by western blot analysis. The release of cyt-*c* was upregulated in the SDT-PBS, SDT-HBSS, ultrasound-PBS and ultrasound-HBSS groups (P<0.05 versus the control-PBS group; Fig. 4). No significant difference in the release of cyt-*c* was identified between the control-HBSS, HMME-PBS and HMME-HBSS groups (P>0.05 versus the control-PBS group; Fig. 4).

### Morphological changes

**Figure 5:**
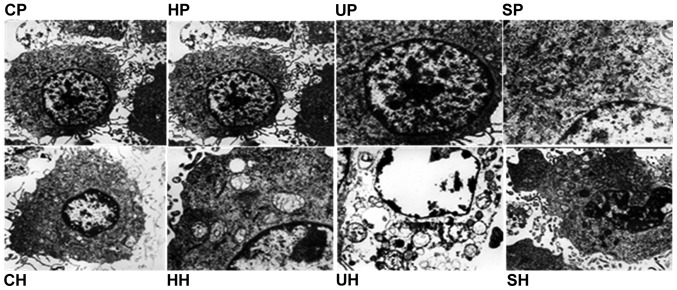
Transmission electron microscopy images of ultrastructural changes in C6 glioma cells (magnification, ×10,000). In the UP, UH, SP and SH groups, nuclear chromatin margination, aggregation, condensation, mitochondrial swelling and vacuolization were observed. CP, control-PBS; HP, HMME-PBS; UP, ultrasound-PBS; SP, SDT-PBS; CH, control-HBSS; HH, HMME-HBSS; UH, ultrasound-HBSS; SH, SDT-HBSS group; HBSS, Hank’s balanced salt solution; HMME, hematoporphyrin monomethyl ether; SDT, sonodynamic therapy.

In order to further investigate the apoptotic effect following SDT treatment, transmission electron microscopy was used to observe the microscopic changes in C6 glioma cells. In the HMME-PBS/HBSS group and the control-PBS/HBSS group, the cell membrane and nuclear envelope were intact, the cytoplasm was rich and mitochondria were integrated. No significant ultra-structural changes were identified. However, the ultrasound-PBS/HBSS group and SDT-PBS/HBSS groups exhibited morphological changes, which were characteristic of apoptosis, including nuclear chromatin margination, aggregation, condensation, swelling and vacuolization of mitochondria (Fig. 5).

## Discussion

The apoptotic effect induced by SDT was dependent on ultrasound intensity, frequency, duration time and sonosensitizers, among others (9,21). Commonly, low-intensity ultrasound is a term describing intensities <3 W/cm^2^ (22,23), and ultrasound frequencies <1 MHz are usually used for drug delivery, blood-tumor barrier opening and ultrasonic therapy (22,24). Buldakov *et al* (24), Bai *et al* (25) and Jeong *et al* (26) observed antitumor effects *in vitro* and *in vivo* induced by SDT with ultrasound intensities of 0.3–2.6 W/cm^2^ and a frequency of 1 MHz. In addition, Zhai *et al* (27) and Ninomiya *et al* (28) showed an inhibitory effect on growth of cancer cells induced by SDT when changing the ultrasound parameters to a frequency of 0.5–1.5 MHz and intensities of 0.4–460 W/cm^2^ *in vitro* (27,28). The lower the frequency and intensity, the higher the cavitation effect and fewer side effects for the healthy surrounding tissue (8,19,20). Therefore, SDT in combination with HMME and the optimized ultrasound parameters of 1.0 W/cm^2^, 0.5 MHz and 60 sec were applied to the C6 glioma cells in the present study. The results revealed the occurrence of apoptosis by flow cytometry and transmission electron microscopy following SDT treatment. The apoptotic rate was significantly increased in the SDT-PBS (49.4±2.6%) group and further increased in the SDT-HBSS (59.9±3.2%) group following SDT, which was higher than that in previous studies whereby apoptotic rate was <40% in C6 glioma cells (3,6,9,18). Accordingly, the low-level ultrasound in combination with HMME may increase the apoptotic effect on C6 glioma cells.

Calcium ions are important in living cells and are the key regulators of cell proliferation and death (15). A crucial requirement for the regulation of cellular functions by cytosolic Ca^2+^ is to maintain a steep concentration gradient between the extracellular and intracellular environments (16). Within the intracellular space, a further Ca^2+^ gradient is established between the cytoplasm and other organelles, including the endoplasmic reticulum and mitochondria (17). Any changes affecting the Ca^2+^ homeostatic balance ultimately influences the fate of cells. The overloaded [Ca^2+^]_i_ accumulated in the mitochondria may alter the outer mitochondrial membrane permeability conversely, leading to the release of cyt-*c* and other apoptotic factors, and eventually apoptosis (10–12). The results of this study revealed that the concentration of [Ca^2+^}_i_ was significantly increased in the SDT group after 30 min when the cells were preincubated in PBS buffer (Ca^2+^-free). This indicated that the [Ca^2+^]_i_ overload was involved in the SDT treatment. In order to investigate the source of the overloaded [Ca^2+^]_i_, the cells were preincubated with HBSS buffer (containing 1.3 mM Ca^2+^). The results revealed a further elevation in [Ca^2+^]_i_ concentration in the SDT-HBSS group. Therefore, the increased [Ca^2+^]_i_ was resultant not only from internal store release, but also from extracellular medium influx during early apoptosis (10,21,24). This result was inconsistent with the previous studies in which the increased [Ca^2+^]_i_ was from the intracellular or extracellular environment (17,18). This contradiction may be due to the different ultrasound parameters and the different types of cells used in the experiment. Liu *et al* (4) proposed that the mechanisms of SDT were influenced by multiple factors, including the nature of the biological model, the sonosensitizer and the ultrasound parameters, among others. In addition, the concentration of [Ca^2+^]_i_ was constant in the SDT-PBS/HBSS and ultrasound-PBS/HBSS groups when the L-type voltage-dependent calcium channel antagonist, nimodipine, was added. This indicated it was ineffective at regulating the concentration of [Ca^2+^]_i_ by SDT treatment. Accordingly, the results indicated that the Ca^2+^ influx may be mediated via mechanisms other than voltage-dependent Ca^2+^ channels, including promotion of membrane permeability and membrane perforation, among others (25); however, further confirmation is required. The results also revealed that the SDT-HBSS group exhibited the highest [Ca^2+^]_i_ concentration and exhibited the highest apoptotic rate. The higher the overload of [Ca^2+^]_i_, the higher the apoptotic rate following SDT treatment. Hence, the increased [Ca^2+^]_i_ from intra- and extracellular sources contributed to the apoptosis induced by SDT.

Previous studies have revealed that ROS production and mitochondrial damage were associated with the apoptosis of C6 glioma cells following SDT (3,17,18,24). In the present study, ROS was increased following SDT using the lower frequency ultrasound. Oxidative damage of ROS induces mitochondrial membrane permeabilization *in vitro* and *in vivo* (11,13–16). When MMP was decreased to a certain extent, cyt-*c*, apoptosis-inducing factor and Smac/Diablo were transmitted from the intermembrane space into the cytosol, which has been defined as an early stage of apoptosis, and then caspase-9 and-3 were activated, carrying out the irreversible apoptotic process (12,21,23). Whether ROS targeted the mitochondria and thereby decreased the MMP in C6 glioma cells was investigated following SDT using the lower ultrasound parameter. The results revealed a significant decrease in MMP and the release of cyt-*c*. Therefore, mitochondrial damage was involved in the apoptotic process under the SDT parameter of the present study. In addition, the mitochondria acted as calcium buffers by sequestering excess calcium from the cytosol. Excessive calcium load to the mitochondria may induce an apoptotic program by stimulating the release of the apoptotic promoting factors from the mitochondrial intermembrane space to cytosol and by impairing their function. The results also revealed that the more ROS production and [Ca^2+^]_i_ were increased, the more the MMP was decreased and the apoptotic rate was increased. Thus, the increased concentration of [Ca^2+^]_i_ and the ROS production followed by the decreased MMP and the release of cyt-*c* may explain the higher apoptotic rate following SDT.

In conclusion, SDT under low-level ultrasound and HMME resulted in an increased [Ca^2+^]_i_ concentration from intracellular and extracellular sources, increased ROS production, a decreased MMP and the release of cyt-*c*. This effect may be applied to treat C6 glioma cells to cause an increased apoptotic effect. Low frequency and low intensity ultrasound with HMME improved the apoptotic effect in glioma cells. The overloaded [Ca^2+^]_i_ was involved in the mechanism by which apoptosis was stimulated and enhanced by SDT.
